# Long-Term Consequences of War Captivity in Military Veterans

**DOI:** 10.3390/healthcare11141993

**Published:** 2023-07-10

**Authors:** Melita Jukić, Luka Malenica, Vanja Đuričić, Jasminka Talapko, Jasmina Lukinac, Marko Jukić, Ivana Škrlec

**Affiliations:** 1 Department of Psychiatry, National Memorial Hospital Vukovar, 32000 Vukovar, Croatia; melita.jukic@ob-vukovar.hr (M.J.); luka94malenica@gmail.com (L.M.); vanja-djuricic@hotmail.com (V.Đ.); 2 Faculty of Dental Medicine and Health, Josip Juraj Strossmayer University of Osijek, 31000 Osijek, Croatia; jtalapko@fdmz.hr (J.T.); iskrlec@fdmz.hr (I.Š.); 3 Faculty of Food Technology Osijek, Josip Juraj Strossmayer University of Osijek, 31000 Osijek, Croatia; ptfosptfos2@gmail.com

**Keywords:** veterans, former prisoners of war, psychological consequences of captivity, predictors of psychological disorders, functioning in marriage and family

## Abstract

Numerous studies on the health and functioning of veterans and former prisoners of war have shown that the experience of war captivity is one of the most difficult human experiences. Captivity is often characterized by extremely difficult and inhumane conditions, as well as exposure to various forms of both psychological and physical abuse. Such traumatic experiences can lead to serious psychological consequences that can last for years, even decades after release from captivity. The aim of this paper is to present a brief overview of research that points to the specifics of wartime captivity and the long-term psychological consequences in veterans of former camp detainees, as well as the consequences suffered by their families and factors that, apart from the intensity of the trauma, contribute to the emergence and persistence of psychological disorders. From the presented research, it can be concluded that former prisoners of the camp represent an extremely vulnerable group of the social community and require long-term appropriate treatment, while the needs of veterans’ families should not be neglected, with the necessity of including spouses and children in psychological and psychosocial treatments.

## 1. Introduction

Traumatic events can happen in any situation, but they are usually isolated incidents with a clear end, and the victim typically has a way out of the problem. In the case of captivity, the victim is exposed to repeated traumatic events and situations from which there is no way out, and the victim is entirely dependent on their abusers, who have tremendous power in these situations over the victim. At the same time, an imprisoned person never knows how long this terrible situation will last. The captors, the abusers, control the entire life of the detainee, deciding what the victim will eat, when and if they will sleep, whether they will wear clothes, and whether they can communicate with anyone. Additionally, a detainee is exposed to various forms of abuse and torture, causing a sense of fear of imminent death, whereby the abuser decides whether to keep the detainee alive. Captivity creates a dominant feeling of fear and loss of all autonomy, often leading, especially in the case of long-term captivity, to a loss of the will to live and a desire to end it. As a result, captivity is considered one of the most difficult human experiences [1]. Numerous studies in the field of psychotraumatology point to the importance of this topic in public health. It is crucial to identify disorders that occur as a result of trauma and to acknowledge the vital role of the social community and support in the recovery process for those affected. This has consistently been confirmed as a significant factor in predicting successful recovery [2]. It is well known from the results of a series of studies that particularly extreme traumatic experiences, such as the experience of captivity, leave a long-lasting mark on the traumatized person, affecting their mental health in various ways. These experiences also affect their relationships with other people, undermining trust in the environment and connectivity with others, including loved ones [3]. When it comes to war veterans who have also experienced captivity after combat, it is regularly emphasized that these two forms of trauma are entirely separate and result in completely different consequences for mental health, which can manifest decades after captivity [4]. Therefore, it is also important that, despite many years having passed since the war’s end, we do not forget those who have been exposed to the most challenging experiences, as victims expect recognition from the community and adequate care. We cannot ignore the experiences of their loved ones and their families either. Research so far shows numerous difficulties faced by families of seriously traumatized veterans, and often, family members, who are expected to provide understanding and assistance, also experience significant problems. Marital relationships and relations with children of heavily traumatized veterans are often significantly damaged [1].

This paper aims to present a brief overview of the research dealing with the experience of captivity and its long-term effects on the mental health of veterans, as well as the consequences for their families. It is important to acknowledge that the effects of war-related psychotrauma can persist long after the war has ended, posing significant social and public health issues. Ongoing research is crucial in psychotraumatology to develop better preventative measures and gain new insights.

## 2. Materials and Methods

The literature search was conducted following the PRISMA Guidelines, utilizing the PubMed, Google Scholar, SCOPUS, and Web of Science databases. The search criteria focused on comprehensive articles in the English language, employing specific keywords, their combinations, and related terms. The inquiry encompassed the concepts of “war captivity” OR “veterans” OR “ex-POW” OR “former prisoners of war” AND “psychological consequences” OR “mental disorder” OR “predictors of mental disorder”, as well as “PTSD in veterans and family”, “veterans marital problems”, “secondary traumatization of veterans” and “veterans’ wives mental disorder.” To optimize the retrieval of pertinent articles, a truncation approach was employed. Initially, 521 articles were identified through a search of titles and abstracts. After a thorough evaluation of the title and abstract, 390 articles were eliminated due to their lack of relevance. Upon scrutinizing the full text of the remaining papers, an additional 15 were excluded as they did not align thematically with the focus of this review. Following the exclusion of 8 duplicates, the study incorporated works that explored the nuances of trauma resulting from war captivity, encompassing investigations on the long-term ramifications and predictive factors contributing to the development and persistence of psychological disorders, as well as the challenges encountered within the families of veterans. A comprehensive array of scholarly works was included, comprising original scientific articles, meta-analyses, literature reviews, and relevant books. In total, a selection of 114 pertinent references was made, comprising 100 original research articles, 3 review articles, and 11 books.

The initial section of the study aimed to provide a thorough depiction of the unique aspects of traumatic experiences during captivity. In this context, older publications were also consulted, chosen based on their efficacy in describing this specific type of traumatization. The subsequent section of the study focused on examining the psychological consequences of captivity in veterans, with a particular emphasis on identifying predictors of mental disorders that contribute to their onset and enduring manifestation. The final section of the study elucidated the psychotrauma-related repercussions impacting the functionality of spousal and familial relationships, along with the secondary traumatization experienced by spouses and offspring of traumatized veterans.

## 3. Results and Discussion

### 3.1. War Captivity—How Is It Different from Other Traumatic Experiences?

Research on war captivity regularly highlights the intensity of the psychotrauma caused by this type of traumatization due to its specific characteristics [5]. It is emphasized that the basic feature of captivity is traumatization directed towards the person, within which the captive is attempted to be humiliated, injured, and broken. Authors highlight a particular type of relationship that arises between the victim (the captive) and the abuser (the captor), which they call a situation of forced control. Various forms of abuse and torture take place on a personal level, and the captive has a sense of complete lack of control over their life. The uncertain situation in which the captive finds themselves intensifies the effect of each individual stressor and leads to a cumulative effect of individual stressful events [1,6,7]. One of the characteristics that distinguishes captivity from most other traumatic situations is that it usually involves prolonged and repeated traumatization that can last for months, and even years. It has been observed that during captivity, individuals go through several phases, each representing a distinct stressor. These phases include capture, detention, the phase of release from captivity and return to their environment, and finally, the phase of reintegration. The authors note that during capture, individuals who were previously in the role of soldiers or combatants must quickly transition to a different role, establish emotional control, confront fears of death, behave in accordance with the new situation, and adjust their behavior in order to survive. Shortly after being captured, the hope of avoiding captivity vanishes, and a sense of despair and disbelief emerges as a result of the series of new events to which the person, now a detainee, is subjected. The individual quickly faces the new reality in which their functioning is forcefully diminished, dealing with the loss of freedom and status. After their release from captivity, those who have survived it encounter a new traumatic experience, that of reintegrating into civilian life [7]. Another characteristic of captivity is the loss of communication with the outside world, the absence of any information, and the loss of connections with loved ones. In captivity, conditions are often extremely difficult, and captives may be exposed to extreme temperatures and unhygienic conditions, and are often deprived of basic needs for food, water, medical care, etc. [1,8]. The stay in solitary confinement, where captives are often placed in a dark room in silence without the ability to communicate with other captives, is described as one of the most traumatic experiences during captivity [9].

Numerous studies describe the experiences of prisoners of war from different wars, such as World War II, the Vietnam War, the Korean War, etc., and the various types of mistreatment and conditions that soldiers were exposed to even before being captured, through their participation in combat activities [10]. Combat trauma can also be intense and involve life-threatening situations, witnessing the suffering of comrades, and killing civilians. However, combat trauma differs in many ways from the situation in which soldiers find themselves after being captured and held in captivity. This traumatization is not directed at the person as soldiers are surrounded by their comrades, usually have the necessary equipment, medical care, and are not deprived of food and water, and most often are not deprived of information and communication [9,11,12].

A number of studies deal with the captivity of soldiers during World War II, and captivity that took place in the Pacific theater, in Japanese war camps, is highlighted as a particularly difficult traumatic situation, characterized by extremely harsh conditions, compared to captivity in the European theater [13,14]. One such study that examines the long-term effects of traumatic experiences in captivity is the one by Sutker and colleagues from 1993. The study included 36 American former prisoners of war and 29 veterans who were not prisoners of war. The vast majority of prisoners were exposed to extreme stressors during their captivity that followed severe combat experiences. Nearly 40% of them were wounded upon capture, many of them experienced neglect of basic bodily needs, and about 90% were exposed to starvation that led to malnutrition. They were housed in overcrowded conditions with extremely poor hygiene. Also, around 90% of them were exposed to extremely high temperatures. About 30% of the prisoners were confined to solitary cells. About 90% of them were forced to march to exhaustion, and the same number experienced physical abuse by beatings. Many witnessed death threats to other prisoners, as well as executions of other prisoners (about 90% of them). They were also subjected to endless, meaningless, and exhausting interrogations, psychological abuse, and about 70% of them were exposed to Allied attacks. [15]. The research conducted more than five decades after the captivity speaks of the horrors suffered by American prisoners of war during World War II. The study included 157 war veterans who were on average 20 years old at the time of their captivity. Some of the participants were held in the European theater, while the others were held in the Pacific theater. The study showed that the experiences of those who were held in the Pacific theater were significantly more brutal [16]. Research on the consequences of captivity among Australian veterans who were held in Japanese war camps also speaks of the harsh conditions and cruelty to which the prisoners were subjected. They were housed in inadequate facilities, had inappropriate clothing, and many suffered from infectious diseases and malnutrition during captivity. They were forced to march to exhaustion and were subjected to various forms of physical and psychological torture, interrogation, and severe punishment. The fact that 30% of the prisoners died during captivity speaks to the conditions and level of cruelty inflicted upon them [17].

In his work on the experiences of captivity during the Vietnam War, Hunter emphasizes that the initial assessments that the group of prisoners of war had almost identical traumatic experiences during captivity proved to be inaccurate. The observed differences in traumatic experiences were based on two important factors: location and time period of captivity. Thus, the author points out that experiences varied depending on whether the prisoners were held in South or North Vietnam, and significantly more severe experiences were had by those who were held captive until August 1969, which is explained by a crucial political moment. He describes the differences in the prisoners’ accommodations, stating that in the south, prisoners were housed in bamboo cages, often chained up, while in the north, they were in masonry buildings, but were subjected to more significant torture. While in the south, their days were spent in cages except when doing cleaning work; those in the north were subjected to significant physical and psychological torture, and even 40% of them spent more than six months in solitary confinement, with 20% spending one to two years and some being locked up for four years [18]. In a paper that examines the consequences of torture endured by Israeli prisoners of war in Yom Kippur War camps, it is noted that they were subjected to starvation, dehydration, severe physical and psychological abuse, electric shocks, beatings, and were kept in small spaces [19].

### 3.2. Psychological Consequences of Captivity

#### 3.2.1. Post-Traumatic Stress Disorder

In the context of the psychological consequences of traumatization, including those resulting from war captivity, the most commonly researched disorder is post-traumatic stress disorder (PTSD). Numerous studies have focused on factors that contributed to its development and persistence over years and even decades after release from captivity, comparing veterans who were camp prisoners with a comparable group of veterans who did not have that experience or with individuals from the general population [20]. However, in addition to PTSD, research also addresses the occurrence of other psychological disorders and predictors of their occurrence [17,21,22]. 

As every captivity has its own specificities and differs, among other things, in the conditions and intensity of traumatic stressors, the applied methods of torture and exhaustion, and the duration of captivity, research results indicate differences in the prevalence of PTSD and other mental disorders among the examined groups. The prevalence of PTSD decades after captivity is, in some studies, associated with the length of stay in captivity. Therefore, depending on the examined veteran population of former prisoners of war, it ranges from 5% to 15% (American veterans of World War II) and up to 80% (Korean War). The time spent in captivity has not been shown to be significant in some studies [13,14,23]. 

The treatment of prisoners has been shown in many studies to be the most significant predictor of the development and persistence of PTSD [24]. Exposure to extreme conditions and cruel treatment, as indicated by significant weight loss in some studies (which also indicates significant vulnerability to traumatization), also leads to psychological consequences that manifest as a high prevalence of PTSD in some groups of prisoners of war [16,25]. 

Based on the occurrence analysis of PTSD, whether it is of a lifetime or current nature, at the time of the research, individual symptoms and clusters of PTSD symptoms, and the association of certain clusters or symptoms with certain types of traumatic events that cause PTSD, as well as the intensity of PTSD in relation to the dominance of certain symptoms, are analyzed. In doing so, the fluctuation of symptoms in the chronic course of PTSD is emphasized, and the symptoms of avoidance behavior, that is, symptoms related to the avoidance of stimuli that remind one of the traumatic event and the circumstances associated with it, are highlighted as the most constant and resistant to therapeutic treatment [26]. Furthermore, in some studies, this group of symptoms is precisely linked to the experience of captivity and exposure to a greater number of traumatic stressors, while symptoms of increased arousal, for example, are associated with combat experience [27,28]. Traumatization during captivity is also associated with pronounced symptoms of emotional numbness, while some studies find a significant correlation between captivity and symptoms of re-experiencing trauma [29,30,31,32].

Studies have shown that avoiding memories of traumatic experiences, especially those that were extremely difficult or involved humiliation and extreme torture, is one of the most prominent symptoms in heavily traumatized veterans, which hinders their recovery [28]. Based on research results, these avoidance symptoms are primarily due to a strong sense of shame about the experiences and humiliation endured. On the other hand, strong feelings of shame are associated with the development of depression, suicidal urges, and psychotic symptoms [16,33].

Research also deals with the feeling of not belonging, and loneliness among war veterans, which is even more pronounced among veterans who have experienced captivity compared to those who have combat experience but were not in captivity. This is explained by the particularities of the captivity trauma, during which not only are prisoners often placed in solitary confinement, but they are also exposed to a specific relationship with their captors that takes place on a personal level, devoid of any empathy and humanity, which will result in a later feeling of insecurity and distrust in interpersonal relationships and lead to loneliness [1]. Some studies deal with the connection between loneliness among veterans and suicidal ideation and suicide attempts [34]. Studies indicate that a significant proportion of former camp prisoners have suicidal thoughts decades after captivity [35,36]. 

A longitudinal prospective study examined the existence of suicidal ideations at three time points (18, 30, and 35 years after the war) in former war camp prisoners compared to veterans who were not camp prisoners [36]. Over time, an increase in suicidal ideation was observed in former camp prisoners, which was more common among them and was also contributed to by PTSD [37].

The occurrence of hallucinatory experiences in relation to the experience of captivity has also been examined [38,39]. In addition, research has shown a correlation between the occurrence of hallucinations and PTSD [40]. For example, avoiding symptoms that lead to social isolation and loss of close relationships with others, as well as the turning of the traumatized person to their inner world, is associated with the development of hallucinatory experiences [41]. Very intense symptoms of trauma re-experiencing can reach a psychotic level, as can symptoms of increased arousal that result in excessive caution and a feeling of being threatened [42,43]. Research involving former prisoners of war from the Yom Kippur War documented significantly more auditory hallucinations decades after captivity among former POWs with PTSD compared to those without PTSD and veterans who were not prisoners of war. In this case, the most significant contribution to hallucinatory experiences was made by intrusive PTSD symptoms [44].

#### 3.2.2. Other Psychological Disorders

Besides the prevalence of PTSD in former prisoners, research also analyzes the occurrence of other mental disorders, usually by comparing participants with PTSD to those without PTSD. While some studies did not find any difference in the occurrence of other mental disorders among the groups with and without PTSD [45], others have shown that other mental disorders are more common in veterans who had or currently have PTSD [46,47]. 

One of the most common comorbid psychiatric disorders is depression, and research emphasizes the importance of recognizing depression and the poorer prognosis and more complicated clinical picture in the case of comorbidity of PTSD and depression [48,49]. The frequent development of depression in severely traumatized camp prisoners has been confirmed in numerous studies, which is, among other things, explained by the phenomenon of loss experienced by these veterans [50,51]. Studies suggest that through inhumane treatment by captors and harsh conditions in captivity, veterans with such painful experiences are faced with a loss of self-esteem, loss of trust in people, and faith in the world around them [52,53]. In addition to depression, the most common comorbid psychiatric disorders were panic disorder, alcoholism, and phobic disorders [47].

Besides the fact that the results of numerous studies link the occurrence of other mental disorders with PTSD, some studies have shown that there is a significant correlation between the experience of captivity, or the intensity of trauma, and the development of certain disorders [54]. At the same time, there was no significant correlation with the development of other disorders. For example, there was no significant difference in the lifetime prevalence of alcoholism in the group of prisoners compared to the general population, while depressive disorders and schizophrenia were more common in prisoners of war [22,55]. 

Studies confirm the vulnerability of former prisoners of war, even fifty years after their imprisonment. In a study that examined the psychological state of former prisoners during the COVID-19 pandemic, it was shown that the consumption of alcohol and marijuana, as well as smoking, increased in this population in order to alleviate their symptoms. This significant vulnerability is explained by deep, severe psychological changes resulting from severe traumatization as part of the interpersonal trauma that is characteristic of imprisonment [56]. In addition to the high prevalence of dissociative disorders in the first years after imprisonment found in studies, a higher prevalence of long-lasting persistent dissociative disorders was documented in former prisoners compared to a control group of veterans who were not prisoners of war [53]. Dissociative states are explained as a mechanism of a kind of escape from thoughts and memories of a near-death experience to which prisoners were exposed through various traumatic situations during their captivity [57]. Some studies do not find a higher prevalence of mental disorders in camp prisoners compared to veterans who were not prisoners. For example, a study that included three groups of camp prisoners and examined the prevalence of mental disorders four or five decades after World War II and the Korean War did not show that the intensity of trauma was a significant predictor of most mental disorders, but it was a significant predictor of the development and persistence of PTSD [58].

#### 3.2.3. Personality Vulnerability, Socio-Demographic Factors, and Social Support as Predictors of Psychological Consequences

Aside from the conditions that prisoners are exposed to during captivity, such as the intensity of traumatic experiences, research also deals with other factors that can affect the outcomes of traumatization. Thus, certain behaviors during captivity and personality traits can contribute to surviving captivity [59]. Personality maturity, emotional stability, higher intelligence, opportunism, as well as optimism, courage, military experience, and belief in a good outcome contribute to survival [59,60,61]. On the other hand, immature, dependent, and passive personality traits with limited adaptive capacities have a greater chance of a poorer outcome and more severe consequences [62]. Studies also deal with the consequences of traumatization in individuals who are focused on the difficulties they face in relation to those who are focused on the emotional states that arise in stressful situations. Studies have shown that individuals labeled as high sensation seekers, characterized by a willingness to take risks and enter risky situations, have a greater chance of a good outcome and easier survival compared to those who are low sensation seekers, and use more favorable defense mechanisms. It has been shown that these two types of personalities have completely different perceptions and subjective experiences of the same reality situations in captivity [63,64,65]. Numerous studies confirm the significance of immediate reactions to a traumatic event, highlighting feelings of shame and anger towards others during or after the trauma, as well as dissociative reactions [66,67]. Research shows that military preparedness and training for extreme situations also reduce the level of distress, and reduce the risk of developing psychological disorders [68].

Some research examines the factors that predict better outcomes for individuals exposed to severe trauma in captivity. For example, a study involving well-trained military personnel, specifically officers of the U.S. military, showed that they had a greater likelihood of coping better in situations of extreme stress and a lower likelihood of developing severe traumatic effects [69]. They were prepared, among other things, for various situations they might encounter if they were to become captive, such as exhaustive interrogations, solitary confinement, clandestine communication, and escape from captivity.

Apart from the factors related to the intensity of traumatic situations, personality characteristics, and immediate reactions to traumatic events, there are several other factors that affect the development, persistence, or chronicity of psychological consequences. Among the sociodemographic factors, the female gender, educational level, age at the time of traumatization, and marital status stand out as one of the most significant predictors of psychological consequences [70]. Adequate social support after release from captivity or return to normal civilian life has been shown to be a crucial factor in the results of numerous studies [71]. Reintegration into normal activities represents an extremely traumatic period after time spent in captivity. Many veterans experience complete social isolation upon returning from war, feel they cannot share their traumatic experiences with others, or encounter negative reactions from their environment, significantly increasing the risk of developing psychological disorders [72,73]. It has also been shown that later life stressors can contribute to the chronicity or onset of symptoms.

The age at the time of captivity is one of the factors that can contribute to the development of PTSD or can be a protective factor. Younger age, lack of life experience, and personality immaturity represent a greater risk for developing PTSD [58]. Studies also indicate that entering older age is a critical period when the risk for intensifying PTSD or reactivating it in already recovered veterans increases [74]. Retirement and poor financial status have also been shown to be predictors of PTSD [30,35]. Higher levels of education have been shown to be predictors of recovery from PTSD in veterans of the Yom Kippur War, including both prisoners of war and veterans who were not prisoners of war [75], For several decades after captivity, other factors contributed to the persistence of depression. Age, education, social support, and socio-economic status were found to be significant, with good socio-economic status being a protective factor in the development of depression [21,76]. A younger age at the time of captivity was associated with greater intensity of anxiety, or a higher prevalence of generalized anxiety disorder several decades after release from captivity in a study that included prisoners from World War II [76]. In addition to a younger age, lower levels of education and lack of social support were also significant predictors of depression several decades after the war in a study that included prisoners from both World War II and the Korean War [21]. Other studies have also shown that lack of social support is one of the most significant predictors of developing PTSD and other psychological disorders [49,77,78,79,80].

In Table 1, 19 studies analyzing the impact of trauma intensity during captivity on long-term psychological consequences were presented, excluding other factors as predictors. Of the 19 studies, 14 included former prisoners of war and veterans without captivity experience as control groups. The examined associations included trauma intensity and the following long-term consequences: post-traumatic stress disorder (PTSD) and posttraumatic stress symptoms; depression; anxiety; suicidal ideation; auditory hallucinations; persistent dissociation; psychiatric and psychosomatic morbidity; PTSD and well-being; subjective sense of benefit; general psychiatric symptomatology; functional problems, and life disability.

Table 2 shows 16 studies examining the association between trauma intensity during captivity and other factors with long-term consequences of traumatization. Apart from former prisoners of war, these studies included veterans without captivity experience as control groups. Among nine studies, former prisoners of war and veterans without captivity experience were included as control groups. The examined factors included sociodemographic factors; personal characteristics and coping with captivity; medical symptoms during captivity; military rank; social support; psychological responses during captivity (9); stressful life events, and subjective quality of life. The long-term consequences examined were PTSD (and PTSS); anxiety; depressive symptoms; negative affect; positive affect; somatic symptoms; interpersonal problems; PTSD and psychiatric comorbidity; retarded activity; subjective quality of life; health-related quality of life; health conditions, and mental health.

### 3.3. Consequences for the Family

One of the most significant predictors of the development and persistence of PTSD and other psychological consequences of traumatization is social support. It has been observed that family and other close individuals represent the best protective factor for psycho-traumatized individuals. Closeness, love, trust, and support that family members provide to the traumatized individual represent the most significant help to them in overcoming difficulties resulting from traumatization [82].

Long-term captivity leads to significant changes in human relationships, and attachment changes resulting from insecurity, anxiety, and loss of trust, even in the closest persons, resulting in the withdrawal and distancing of the traumatized person from their loved ones, who are expected to provide support and help at the same time [81,83,84]. Intensive traumatic experiences with severe psychological consequences, such as those experienced by veterans and former prisoners of war, make it difficult to receive support but also to provide support to the traumatized person, often resulting in serious difficulties in marital and family relationships, leading to psychological consequences for both spouses and offspring [85]. PTSD is often associated with difficulties in intimacy, trust, expressing emotions, aggressive behavior, loss of interest, and distancing from the environment, all of which contribute to poor marital adjustment and difficulties in the adjustment of the spouses of veterans with PTSD [86,87]. Research has shown that less severe symptoms of post-war stress make it easier to provide support to traumatized individuals [88].

The importance of the influence of the experience of confinement and PTSD on marital relationships is also evident in the results of studies that find a higher frequency of divorce in veterans who were prisoners of war compared to those who were not [89]. Marital adjustment is a complex concept that denotes a process that changes over time and refers not only to satisfaction within the marital relationship but also to the collaboration of partners, agreement on important life decisions, and the expression of affection, closeness, and emotions between partners [90,91]. Studies also indicate differences in marital adjustment among those with chronic PTSD compared to those with sudden-onset, delayed PTSD, where delayed PTSD in former prisoners of war is associated with lower levels of marital adjustment [92]. Often, within a marriage that is burdened with severe psychotrauma, the phenomenon of overprotectiveness arises. This phenomenon is characterized by the excessive protection of the traumatized veteran by their spouse, as well as the complete adaptation of family life to their needs. Such a relationship can lead to significant distress in the spouse, who takes on the role of protector for the veteran and their children from stressful family situations associated with symptoms of PTSD. In this process, wives often receive nothing in return and often develop feelings of helplessness and insecurity, which is further contributed to by the frequent loss of contact with others, or social isolation, of both the veteran with PTSD and their spouse [93,94]. As a result, wives of veterans often find themselves being judged by their surroundings and developing a sense of abandonment. The environment often condemns the display of anger and similar emotions by the veteran’s spouse, interpreting them as betrayal of the veteran, thus making it impossible for them to express negative emotions, as they receive no understanding for the suffering and difficulties of their own [95].

Emotional engagement, empathy, compassion, and taking on all obligations and responsibilities without satisfaction and the possibility of relaxation can lead to a phenomenon of “compassion fatigue” in veteran’s spouses, according to Figley [96]. Despite taking on all family responsibilities, wives of former prisoners often take a secondary position in the family and marriage “because they feel they are not entitled to equality with their hero spouses” [97].

In veteran’s spouses, as well as in other close family members, secondary traumatization can occur, which means that symptoms similar to those of the primary traumatized person may develop, such as symptoms of avoiding stimuli that remind them of trauma, symptoms of increased arousal, and sleep difficulties. Studies show a clear correlation between PTSD symptoms in veteran spouses and PTSD symptoms in veteran prisoners of war [98,99]. Wives of former prisoners and other veterans may also develop depression, anxiety, and strong feelings of guilt, caused by living with a severely traumatized spouse who is often only physically present but emotionally distant and excluded from everyday life. Relationships are therefore burdened with a number of uncertainties about the functioning of marriage and family [100].

Severe trauma and its consequences inevitably affect not only marital relationships but also relationships of traumatized individuals with their children, often making them dysfunctional in their parenting role. Veterans with PTSD have no desire or interest in relationships with their children, or they exhibit overly controlling behavior [101,102]. This leads to extremely complex relationships that can leave a mark on the psychological development of children, where emotional numbness, absence, and distancing of the veteran contribute the most [103].

Research has shown that some veteran’s children were not aware of the causes of their father’s behavior until adulthood, but they remember his mental disorders and the disturbed dynamics of family relationships, where everything was subordinated to the father, and the family atmosphere was marked by fear, caution, and feelings of guilt [104]. Growing up with a father with pronounced attachment insecurities is marked by feelings of insecurity, loneliness, rejection, mistrust, and the development of a negative self-image [105].

Various mental disorders and disturbances can occur in children of veterans with PTSD, such as depression, anxiety, hyperactivity, and difficulties in social functioning. Some studies identify violent behavior of veterans as the cause of behavioral disorders and academic failure more than PTSD itself [106]. Research shows that adult offspring of former prisoners of war have a higher rate of PTSD, depression, anxiety, and attachment insecurities compared to offspring of veterans who were not prisoners of war [107]. The importance of psychotrauma in family dynamics and its impact on children is demonstrated by research, the results of which show that even in cases where a traumatized person has not developed a mental disorder and does not speak about the trauma experienced, trauma can still be transmitted to the child through parenting methods and growing up in a family atmosphere where a “secret” is kept and something important is not talked about [108].

Table 3 focuses on the consequences experienced by families of war veterans. The participants in the studies were veterans and their spouses; veterans and their children; women married to veterans; veterans and their families; spouses and children of veterans, and children of veterans. The research topics covered the quality of intimate relationships; problems in marital and family adjustment, parenting skills, and violent behavior; spouses’ perception of marital relationships; predictors of divorce; secondary traumatization in veterans’ spouses; paranoia in spouses and children of veterans; psychological difficulties in children of veterans; the experience of captivity and its consequences from the perspective of veterans’ spouses; the association between veterans’ aggressiveness and aggressive behavior in children; the quality of relationships between veterans and their children, and secondary traumatization in children of veterans.

The trauma experienced by veterans also affects the parents of traumatized soldiers, although such studies are significantly less frequent than those related to the secondary traumatization of veterans’ wives or children [99]. In a study that deals with the consequences of traumatization in Vietnam veterans and the impact of their war experiences on their later family life and functioning, the importance of life educators and therapists is emphasized, along with the need for enhanced interventions at the family level [109]. Studies exploring motherhood in the context of various military conflicts are interesting. Udi Lebel, for example, discusses the victimological militarism of mothers of soldiers who were killed in action. Their tragedy and loss bring them an important position in society by perpetuating a culture of martyrdom, which can hinder recovery. Conversely, survivors who are severely traumatized, including former prisoners of war, are considered less valuable and rank lower on the heroism scale, resulting in reduced possibilities of social recognition, support, and respect [110].

The issue of respecting the families of traumatized veterans can also be viewed from a different perspective. If the family is considered the foundation of society, it becomes evident that, in the context of involving young people in the military forces, the importance of the family must not be neglected. It is within the family that attitudes are formed, and the groundwork for readiness to join military units is laid. The research authors discuss the relationship between the state, society, family, and parenting in the context of the family’s sacrifice during wartime conflicts. They highlight the different positions of families of fatalities and survivors, as well as the varying ways in which families process their losses. Some families may grieve privately, while others, as many have undertaken, choose to engage socially and politically, actively participating in the struggle to bring their family members back from the battlefield [111].

## 4. Limitations to the Study

Limitations of this review should be acknowledged. Firstly, it is important to note that the scope of this review does not encompass articles that specifically focus on civilian detainees or prisoners. Secondly, this paper does not delve into the experiences of perpetrators of abuse, nor does it include individuals who provided support or witnessed the abusive acts. Despite the established responsibilities outlined in the Geneva Conventions, regrettably, violations of these conventions continue to persist. One noteworthy occurrence exemplifying this is the documented events described in General Taguba’s report, which unveils the mistreatment of prisoners in Iraq. Published in 2004, this report, titled after its author, brings to light these unsettling actions [112]. In the context of preventing abuse in confinement and recognizing the subsequent repercussions for detainees and their families, it is crucial to underscore the significance of upholding the Geneva Conventions and the ensuing obligations they entail. It is imperative to abide by these obligations not only in the treatment of prisoners but also in ensuring adequate sanitary and health conditions within the bounds of captivity [113]. Furthermore, when discussing confinement, we must not disregard the importance of educating officers on the proper treatment of prisoners and their indispensable duty to safeguard detainees [114]. Lastly, this review paper does not delve into detailed descriptions of abuse among detainees or delve into the consequences faced by detainees who participated in or witnessed such abuse. It is essential to recognize these limitations in order to understand the specific boundaries of this study and the areas that were not explored in depth.

## 5. Conclusions

Numerous studies of the effects of trauma on war veterans have shown that the psychological effects of extremely severe traumatic experiences, such as the experience of captivity, can persist decades after exposure to traumatic events. It has also been shown that the occurrence of psychological disorders is influenced by a number of factors in addition to the intensity of the traumatic experience, with the importance of social support being particularly salient. In addition to concerns for veterans’ health, it is important not to forget family members who are also affected by living with a severely traumatized person, as numerous consequences have been documented in these cases. Further research on this topic is needed to determine the best methods for preventing and treating mental health disorders in this vulnerable population.

It would be interesting to research veterans’ families in the context of secondary traumatization, comparing socially active families with those that were not involved. Additionally, conducting research on the consequences of abuse in detention based on the testimonies of survivors would undoubtedly be beneficial. Moreover, exploring positive events during times of war would also be valuable to investigate.

## Figures and Tables

**Table 1:** Impact of captivity on long-term consequences.

Authors	Subjects	Research Area	Results
Dekel et al. (2014) [4]	275 former prisoners of war (ex-POWs) and 219 matched combatants (controls) in Yom Kippur War (1973).	The prevalence of depressive symptoms and PTSD symptoms was assessed in both groups at three different time points over a period of 17 years.	Depression and PTSD appear to be distinct long-term outcomes of traumatic stress, influenced to some extent by the severity of the trauma experienced. POWs exhibited higher rates of depressive symptoms and PTSD compared to the control group.
Sutker and Allain (1996) [13]	326 POWs of the World War II (WWII) European and Pacific theater and Korean Conflict (KC), and in 214 combat veterans of both wars and KC POW survivors, and 112 combat veterans of the same military operations who were not captured.	POWs’ trauma severity, captivity weight loss, and captivity duration and prevalence.	KC and WWII Pacific ex-POWs reported the highest levels of trauma and exhibited the highest rates of lifetime and current mental disorders, including PTSD. Notably, POW subgroups displayed greater psychopathology compared to combat veterans. The prevalence of PTSD was consistently high across all subgroups and frequently co-occurred with other mental disorders, indicating the enduring and wide-ranging impact of combat and POW experiences on mental health.
Sutker and Allain (1993) [15]	36 POW survivors and a group of 29 combat veterans were compared approximately 40 years after the war (WWII Pacific theater).	The impact of traumatic experiences on anxiety, mood states, and the prevalence of post-traumatic stress disorder (PTSD), anxiety disorders, and depressive disorders.	Both groups showed a high occurrence of anxiety and depressive disorders. However, there were discrepancies in the rates of PTSD diagnoses. Among the POW survivors, 70% met the criteria for a current diagnosis of PTSD, and 78% met the criteria for a lifetime diagnosis. In contrast, the combat veterans had lower rates, with only 18% meeting the criteria for a current diagnosis and 29% for a lifetime diagnosis of PTSD.
Rintamaki et al. (2009) [16]	157 American military veterans who were former WWII POWs (the European and Pacific theaters).	POW experiences, effects on subsequent psychological and physical well-being, and ways in which these experiences shaped major decisions in their lives.	WWII POWs from Europe and the Pacific reported long-term emotional impacts from their captivity. Both groups had high rates of reflection, dreaming, and flashbacks about their POW experiences. Pacific theater POWs had higher current rates than in the past. After retirement, both groups ruminated more on their POW experiences; 16.6% met clinical PTSD criteria, with Pacific theater POWs three times more likely (34%) than European theater POWs (12%). Traumatic memories and clinical PTSD persist for up to 65 years after captivity. Rumination, flashbacks, and nightmares may increase after retirement, especially for Pacific theater POWs.
Tennant et al. (1986) [17]	170 Australian POWs of the Japanese and 172 non-POW controls (WWII).	Evidence of chronic psychiatric and psychosomatic morbidity over the 40-year period following the war.	POWs did not show a higher likelihood of psychiatric admissions compared to non-POWs, and fewer of them had multiple psychiatric admissions.
Tsur et al. (2017) [19]	59 Israeli ex-POWs who experienced severe torture in captivity, and 44 matched controls (Yom Kippur War).	Examination of chronic pain personification in torture survivors and post-traumatic stress disorder (PTSD) (at 18, 30, and 35 years after captivity).	Ex-POWs show higher levels of torturing personification but no differences in concrete description of chronic pain compared to controls. Torturing personification intensity varies with PTSD trajectories. Sequential mediation analysis reveals that PTSD at T2 and T3 mediates the link between torture and torturing personification.
Myers et al. (2005) [25]	102 male subjects who were in combat either in WWII (96) or in the Korean Conflict (6) and who had been held captive as a prisoner of war for a minimum of 3 months.	Relationship between duration captivity and weight loss during captivity and current PTSD symptoms.	The extent of weight loss experienced during imprisonment significantly correlates with the intensity and frequency of current PTSD symptoms in ex-POWs, even after more than five decades. However, there is no relationship between the duration of POW imprisonment and current PTSD symptoms.
Neria et al. (2000) [31]	164 POWs and 189 matched combatants of the 1973 Yom Kippur War.	Intensity of post-traumatic stress reactions, general psychiatric symptomatology, and problems in functioning at home and at work.	Ex-POWs showed higher rates and intensity of post-traumatic stress reactions, increased psychiatric symptoms, and more severe functional impairments at home, work, and in the military compared to the control group.
Henigsberg et al. (2001) [32]	136 PTSD patients exposed to war-related traumatic experiences divided into four groups: 79 veterans, 18 former prisoners, 15 victims of rape, and 24 refugees from Bosnia and Herzegovina.	Investigation of trauma relation to specific symptom patterns in patients with PTSD.	There was compelling evidence suggesting that the characteristics of stressors have a significant impact on the range and quantity of intrusive, avoidance, and arousal symptoms displayed.
Stein et al. (2017) [34]	Two groups of Israeli veterans of the 1973 Yom Kippur War, 163 ex-POWs, and 185 matched non-captive veterans were assessed 18 (T1) and 30 (T2) years after the war.	PTSD symptoms and loneliness have been individually assessed.	Analyses indicated that compared with war, captivity was implicated in worse PTSD, which was implicated in worse loneliness. Loneliness, however, was not directly affected by the type of trauma.
Hunt et al. (2008) [35]	328 former U.S. military personnel held as POWs (WWII and Korean and Vietnam Wars).	Identification of aspects of POWs’ incarceration which are associated with later life disability status.	There are notable connections between disability in later life and various POW experiences such as torture, witnessing torture, solitary confinement, forced marches, dysentery, pellagra, vitamin deficiencies, scabies, depression, and suicidal ideation.
Zerach et al. (2014) [36]	Two groups of male Israeli veterans from the 1973 Yom Kippur War were examined: ex-POWs and comparable veterans who were not taken captive.	Assessing suicidal ideation (SI) among ex-POWs and its associations with post-traumatic stress disorder (PTSD) symptoms over a 35-year period (T1 18 (1991), T2 30 (2003), and T3 35 (2008) years after the war).	Compared to control veterans, ex-POWs reported elevated levels of suicidal ideation (SI) at T2 and T3, along with an increasing trend in SI over time. Among ex-POWs, PTSD symptoms at T1 contributed to the acceleration of SI rate of change over time. Furthermore, PTSD symptoms had a significant impact on SI concurrently, surpassing the effects of SI trajectories.
Zerach et al. (2017) [37]	189 Israeli ex-POWs and 160 comparable combatants participated in a 17-year longitudinal study with three waves of measurements following the 1973 Yom Kippur War (T1: 1991, T2: 2003, T3: 2008).	Evaluation of long-term trajectories of post-traumatic stress symptoms (PTSS) in ex-POWs and similar veterans, as well as the predictive role of hardiness and sensation seeking in PTSS trajectories.	Long-term PTSS trajectories vary among veterans and ex-POWs, with a higher prevalence of chronic and increasing symptom patterns observed in ex-POWs. Ex-POWs, as an at-risk population, exhibit exacerbated PTSS trajectories associated with the personality construct of hardiness.
Crompton et al. (2017) [38]	99 ex-POWs from the 1973 Yom Kippur War with and without PTSD and a group of 103 comparable veterans.	The prevalence of auditory hallucinations in trauma survivors and its association with post-traumatic stress disorder (PTSD) symptoms, over time, in 1991 (T1) and 2003 (T2).	Ex-POWs with PTSD exhibited higher levels of auditory hallucinations at T2 and experienced an escalation in hallucinations over time, in contrast to ex-POWs without PTSD and combatants who were not held captive. The overall PTSD score at T1 predicted an increase in auditory hallucinations between T1 and T2, but not the other way around. Among the PTSD symptoms, intrusion symptoms made a distinct contribution in relation to avoidance and hyperarousal symptoms.
Ohry et al. (1994) [39]	164 POWs and 190 controls (Yom Kippur War).	Long-term morbidity (18 years), psychophysiological complaints, and illness-related behaviors and association with characteristics of captivity.	POWs exhibited significantly higher psychophysiological complaints compared to the controls, and these complaints were significantly associated with symptoms of post-traumatic stress disorder (PTSD). The level of impairment experienced by individuals was connected to captivity-related factors.
Engdahl et al. (1998) [47]	262 American former prisoners of war (WWII and Vietnam War).	Investigation of comorbidity, time of onset, and the relationship of trauma severity to complicated versus uncomplicated PTSD.	PTSD typically arises shortly after experiencing trauma. Lifetime PTSD is linked to a higher risk of lifetime panic disorder, major depression, alcohol abuse/dependence, and social phobia. Current PTSD is associated with an increased risk of current panic disorder, dysthymia, social phobia, major depression, and generalized anxiety disorder.
Zerach et al. (2014) [57]	Israeli veterans from the 1973 Yom Kippur War: ex-POWs and comparable veterans who were not taken captive (at three time points: T1 18 (1991), T2 30 (2003), and T3 35 (2008) years after the war).	Assessing the impact of war captivity on persistent dissociation (PD) and the longitudinal relations between captivity stressors, post-traumatic stress disorder (PTSD), and PD.	Ex-POWs with PTSD displayed elevated levels of PD compared to ex-POWs and non-POW veterans without PTSD at T3. Additionally, PTSD symptoms at T1, T2, and T3 mediated the connection between captivity and PD at T3. PD was linked to loss of emotional control, detachment reactions to captivity, and post-traumatic intrusion symptoms.
Sladge et al. (1980) [60]	221 repatriated POWs of the Vietnam War still on active duty, and 341 matched controls in 1976.	Individual experiences of the stress and frustration and the long-term consequences of the war imprisonment experience.	Subjective sense of having benefited from the experience of war imprisonment was positively correlated with the harshness of the experience.
Solomon et al. (2008) [81]	103 ex-POWs and 106 comparable control veterans from the 1973 Yom Kippur War.	Post-traumatic symptoms, attachment anxiety, and attachment avoidance were assessed at two points in time, 18 years (T1) and 30 years (T2) after the war.	Ex-POWs exhibited higher post-traumatic symptoms compared to controls at both measurement points, and these symptoms increased solely among ex-POWs from T1 to T2. Attachment anxiety and attachment avoidance increased over time among ex-POWs, while they slightly decreased or remained stable among controls. Furthermore, the rise in attachment anxiety and avoidance was positively linked to the increase in post-traumatic symptoms in both study groups.

**Table 2:** The influence of captivity and other factors on long-term consequences.

Authors	Subjects	Research Area	Results
Park et al. (2012) [5]	292 repatriated POWs (Vietnam War).	Extent to which demographic, captivity stressors, and indicators of mental health at repatriation predicted long-term mental health outcomes (PTSD, anxiety, and depressive symptoms).	The age at capture and post-traumatic stress symptoms upon repatriation were predictors for all three long-term mental health outcomes. Additionally, physical torture was associated with long-term post-traumatic stress symptoms. These findings emphasize the enduring impact of captivity and the remarkable resilience demonstrated by individuals.
Neria et al. (2014) [9]	136 POWs who were captured by the Egyptians and 28 who were imprisoned by the Syrians (18 years after the Yom Kippur War).	Sociodemographic background, battlefield stressors, captivity severity (weight loss in captivity, physical and psychological abuse, severity of the humiliation). Psychological responses during captivity and social support at homecoming.	Psychological responses during captivity were identified as the primary factors influencing the mental health of POWs. Additionally, the significance of education and ethnic status followed, with the severity of captivity also playing a role.
Engdahl et al. (1991) [21]	989 U.S. ex-POWs (WWII and the Korean War).	Sociodemographic status and medical symptoms during captivity, weight loss, and level of social support related to later levels of adjustment. Higher negative affect (NA), somatic symptoms and retarded activity (SO), interpersonal problems (IP), and lower positive affect (PA).	Younger age at capture, lower education level at capture, increased medical symptoms during captivity, and reduced social support were predictors of long-term maladjustment. Age at capture did not predict subjective well-being (SO), and captivity symptoms did not predict positive affect (PA). However, elevated levels of medical symptoms during captivity and low social support were especially predictive of later increased negative affect (NA) and subjective well-being (SO) scores.
Jukić et al. (2022) [29]	Croatian Homeland War veterans—264 combat veterans (116 had been held as prisoners of war and the remaining 148 were the control group) with diagnosis of PTSD.	The impact of sociodemographic factors, intensity of trauma, and social support on PTSD intensity and psychiatric comorbidity	Ex-POWs experienced a significantly greater number of traumatic events and had a higher prevalence of psychiatric comorbidities overall. The incidence of acute and transient psychotic disorders, generalized anxiety disorders, and psychological and behavioral factors related to disorders or diseases classified elsewhere was notably higher among ex-POWs.
Lončar et al. (2014) [30]	184 male participants who have survived war imprisonment during the Croatian Homeland War.	Examination of the role of socio-demographic characteristics, war experiences, and subjective quality of life in the prediction of three clusters of PTSD symptoms.	Traumatic war experiences predicted avoidance symptoms, while traumatic war experiences and subjective quality of life predicted hyperarousal symptoms. Additionally, traumatic war experiences, material status, and subjective quality of life were predictors of intrusion symptoms.
Jukić et al. (2019) [46]	Two groups of veterans from the Homeland War in Republic of Croatia (45 ex-POWs, 45 which were not imprisoned—control group). All participants were diagnosed with PTSD and had combat experience.	The health-related quality of life (HRQoL) in ex-POWs affected by PTSD, regarding the intensity of PTSD symptoms, sociodemographic characteristics, and somatic comorbidity. Identification of poor HRQoL predictors.	PTSD was associated with the HRQoL, whether the veterans were ex-POWs or not. Low socioeconomic status has proved to be the most significant predictor of poorer HRQoL.
Engdahl et al. (1997) [58]	262 US WWII and Korean War ex-POWs.	The long-term impact of traumatic experiences within the context of post-traumatic psychopathology.	Over half of the men (53%) met criteria for lifetime PTSD, and 29% met criteria for current PTSD. The most severely traumatized group (POWs held by the Japanese) had lifetime rates of 84% and current rates of 59%. Among those with current PTSD, 55% had uncomplicated PTSD without other concurrent disorders. Furthermore, 34% of those with lifetime PTSD had PTSD as their sole diagnosis. Age at capture, trauma severity, and postmilitary social support moderately predicted PTSD and weakly predicted other disorders.
Solomon and Dekel (2005) [53]	209 Israeli veterans of the 1973 Yom Kippur War (103 ex-POWs and 106 controls) 18 and 30 years after release from captivity.	PTSD changes over time, and the contribution of captivity severity (objective and subjective), sociodemographic variables, and psychological appraisal and coping with captivity to predicting PTSD.	Among the ex-POWs, 23% met criteria for PTSD and were 10 times more prone to psychological deterioration in the 12-year period between the two assessments, compared to controls. Nearly 20% of ex-POWs who did not meet PTSD criteria in 1991 now met criteria in the current assessment, in contrast to nearly 1% of the controls. Younger age at captivity, loss of emotional control, higher subjective appraisal of suffering during captivity, and a greater number of PTSD symptoms in the 1991 assessment were predictive of current PTSD.
Solomon et al. (2023) [56]	120 Israeli ex-POWs and 65 matched veterans of the 1973 Yom Kippur War after 18 (T1), 30 (T2), 42 (T3), and 47 years after the war.	The implication of exposure to distant trauma of war captivity, stressful life events across the life span, and PTSD trajectories and current PTSD.	The enduring impact of severe traumatic stress during young adulthood and subsequent trajectories of PTSD are evident in increased substance use among the elderly population.
Solomon et al. (2014) [64]	154 Israeli ex-POWs and a matched control group of 161 combat veterans 18 and 35 years after participation in the Yom Kippur War.	Assessed the long-term impact of war captivity on mortality and various health aspects and evaluated the potential mediating role of post-traumatic stress disorder (PTSD) and depressive symptoms.	Captivity was linked to premature mortality, increased health-related conditions, and poorer self-rated health. The relationship between war captivity and self-rated health was mediated by PTSD and depressive symptoms, while the relationship between war captivity and health conditions was partially mediated by these symptoms, with the effects being more pronounced with age. Aging ex-POWs who develop psychiatric symptoms should be recognized as a high-risk group entering a vulnerable stage of life.
Solomon et al. (1995) [65]	164 Israeli ex-POWs and 184 comparable controls 18 years after their participation in the Yom Kippur War.	Implication of both sensation seeking and the subjective appraisal of captivity in the long-term adjustment of ex-POWs.	Ex-POWs with low sensation-seeking tendencies displayed higher levels of PTSD symptoms, more severe psychiatric symptoms, and greater intensity in intrusive and avoidance behaviors. High and low sensation-seeking POWs also differed in their emotional experiences when taken prisoner, subjective evaluation of suffering in prison, coping strategies employed during captivity, and emotional states experienced.
Sutker and Allain (1995) [68]	33 WWII aviators who were held as prisoners of war.	Impact of POWs’ demographic characteristics, military rank, and personal resources on rates of PTSD and resilience to captivity.	The results revealed higher MMPI profile patterns than anticipated based on previous research with pilots, along with a prevalence of current and lifetime PTSD at 33%, believed to be associated with the trauma of being a POW. WWII aviator POW survivors exhibited greater resilience to the effects of captivity compared to non-aviator WWII POW survivors of similar age, who generally possessed fewer advantages in education, military rank, and personal resources.
King et al. (2011) [69]	241 U.S. Naval aviators, Army soldiers, and Marines who were held as prisoners of war during the Vietnam War.	Examination of relations between personal and military demographics and captivity stressors with mental health outcomes (post-traumatic stress symptomatology (PTSS), general distress, and interpersonal negativity).	Hierarchical multiple regression analyses revealed that being an officer acted as a protective factor in the relationships between physical torture and PTSS, psychological torture and PTSS, and psychological torture and interpersonal negativity.
Solomon and Dekel (2005) [74]	209 Israeli veterans of the 1973 Yom Kippur War (103 ex-POWs and 106 controls).	The psychological responses to captivity 18 and 30 years after release from captivity (rates of PTSD, changes in PTSD over time, and the contribution of captivity severity, sociodemographic variables, and psychological appraisal and coping with captivity to predicting PTSD.	Younger age at the time of captivity, experiencing a loss of emotional control and higher subjective perception of suffering during captivity, and having a greater number of PTSD symptoms in the 1991 assessment predicted the presence of PTSD in 2003.
Jukić et al. (2022) [79]	259 Croatian Homeland War veterans diagnosed with PTSD, with at least 6 months of combat experience. Among them, 90 subjects had also experienced imprisonment in enemy prison camps (at least 1 month of captivity).	Investigation of the effect of self-perceived social support on the intensity of PTSD symptoms and mental health-related quality of life (MHRQoL) in veterans more than two decades after exposure to trauma.	The obtained results showed that veterans who had a more positive perception of social support after the events of the war had less intense PTSD symptoms and better MHRQoL.
Bachem et al. (2021) [80]	149 ex-POWs and 107 combat veterans (1991, 2003, 2008, and 2015).	Evaluation of heterogeneity of changes over time and investigation of the contribution of trauma exposure (combat vs. war captivity), hardiness, and social support for depression trajectories.	Four distinct trajectories were identified: “resiliency” (62.8%), “delayed onset” (25.1%), “exacerbation” (6.2%), and “chronicity” (5.9%). The resilient group predominantly comprised combat veterans, while the clinical groups primarily consisted of ex-POWs. Lower levels of hardiness and social support were associated with more unfavorable trajectories.

**Table 3:** Consequences of the war trauma of veterans for their families.

Authors	Subjects	Research Area	Results
Riggs et al. (1998) [85]	26 heterosexual couples in which the veterans had PTSD were compared to 24 couples in which the veteran did not have PTSD.	Quality of the intimate relationships of male Vietnam veterans.	Approximately 70% of veterans with PTSD and their partners experienced clinically significant levels of relationship distress, while the percentage was around 30% for non-PTSD couples. PTSD veterans and their partners reported greater relationship problems, intimacy difficulties, and a higher likelihood of considering separation and divorce compared to non-PTSD veterans and their partners. The level of relationship distress correlated with the severity of PTSD symptoms in veterans, particularly emotional numbing symptoms.
Jordan et al. (1992) [86]	Nationally representative sample of 1200 male Vietnam veterans and the spouses or co-resident partners of 376 of these veterans.	The presence of PTSD and items affecting family and marital adjustment, parenting problems, and violence.	Families of male veterans with current PTSD exhibited significantly higher levels of severe and widespread issues in marital and family adjustment, parenting skills, and violent behavior compared to families of male veterans without current PTSD.
Dekel et al. (2005) [87]	9 wives of veterans with PTSD.	Examination of the marital perceptions of veterans’ wives and how the lives of these women largely revolved around their husband’s illness.	The wives experienced a continuous tension between becoming deeply connected with their husbands and striving to maintain their independence. Furthermore, they acknowledged positive aspects of the marital relationship that provided them with strength for present and future coping.
Nice et al. (1981) [89]	Study I: 101 Navy POWs repatriated from Vietnam (RPWs) and 100 in a comparison group. Study II: 29 RPW families and 38 RPW comparison families.	Marital stability and perceptions of marital adjustment and family environment were investigated among Navy prisoners of war RPWs and a Navy comparison group.	The rate of divorce after repatriation was significantly higher among the RPW group compared to the comparison group. Predictors of divorce among RPW families included having fewer children, longer duration of captivity, and religious affiliation. When comparing perceptions of marital adjustment and family environment, no significant differences were found between a subset of RPW and comparison families who remained together.
Levin et al. (2016) [92]	66 wives of ex-POWs with PTSD, and 37 wives of ex-POWs and 55 wives of combat veterans without PTSD symptoms.	Evaluation of the implications of war captivity and ex-POWs’ PTSD, and PTSD trajectory on their wives’ marital adjustment, adjusting for their secondary traumatization.	The study’s findings indicate that wives of ex-POWs with PTSD reported lower levels of consensus, affection, and overall marital adjustment compared to the wives of controls, even after accounting for secondary traumatization. This suggests that both the experience of captivity and PTSD have a negative impact on wives’ marital adjustment, extending beyond the individual effects of secondary traumatization.
Glassman et al. (1987) [84]	A Vietnam veteran and his family.	Case report: Expression of veteran’s paranoid schizophrenia involved delusions and hallucinations relating to Vietnam, and his wife and children’s paranoia.	Wife and children shared veteran’s paranoia.
Hall and Simmons (1973) [95]	POWs’ wives and children.	Psychological issues centered on themes of desertion, ambiguity of role, repressed anger, sexuality, censure, and social isolation.	Severe and progressive psychological and psychophysiological symptoms are frequently observed, with common manifestations including separation anxiety, role distortion, and sleep disorders among children. Male children tended to experience these effects more significantly than their female counterparts.
Lieblich (1997) [97]	Israeli POWs’ wives.	The cases of Israeli POWs are presented from their wives’ points of view.	Their narratives depict the abrupt separation, the challenges faced during the prolonged and uncertain waiting period, and their emotional responses upon reunion. These voices contribute to the construction of the social role attributed to the wife of a POW. Unfortunately, society often overlooks or underestimates the courage and suffering endured by these wives, despite the fact that they undergo their own trauma and struggle to adapt to the political and personal events that shape their lives.
Haley (1984) [101]	Two case examples involving 26- and 27-year-old Marines with PTSD symptoms.	Investigation of the activity and aggression of the preschool child reawakens the painful effects of combat aggression and sadism in the Vietnam combat veteran.	Efforts to manage aggression in both the veteran and his children can potentially result in ineffective coping strategies and the emergence of symptoms within the veteran, the child, or the entire family system.
Ruscio et al. (2002) [102]	66 male Vietnam veterans and their children.	Examination of the differential pattern of associations between the symptom clusters of PTSD and the perceived father–child relationships	The cluster of emotional numbing exhibited a significant association with the perceived quality of all relationship domains, suggesting that it is the aspect of PTSD most strongly connected to interpersonal difficulties in war-zone veterans.
Dekel and Goldblatt (2008) [103]	Review.	Review of the literature on intergenerational transmission of PTSD from fathers to sons in families of war veterans.	This review highlights the current paucity of knowledge regarding family members and extrafamilial systems that may contribute to intergenerational transmission of PTSD or to its moderation.
Zerach and Aloni (2015) [107]	98 Israeli ex-POWs’ children and 90 controls’ children whose fathers fought in the 1973 Yom Kippur War.	Examination of the secondary traumatization symptoms and parental bonding among adult children of ex-POWs’ children that were compared to adult children of comparable veterans (controls’ children) and the mediating role of parental bonding and exposure to stress in the association between group and secondary traumatization symptoms.	Four decades after the war, living with ex-POWs is linked to the psychological well-being of their children. Ex-POWs’ children exhibit more symptoms of secondary traumatization and lower levels of paternal care compared to children of controls. The association between the research group and secondary traumatization is mediated by exposure to stress caused by fathers’ behaviors and the level of paternal care.

## Data Availability

Data are contained within the article.
